# The significant role of post-pairing male behavior on the evolution of male preferences and female traits

**DOI:** 10.1038/s42003-021-02961-x

**Published:** 2022-01-10

**Authors:** Nan Lyu, D. Justin Yeh, Huw Lloyd, Yue-Hua Sun

**Affiliations:** 1 Ministry of Education Key Laboratory for Biodiversity and Ecological Engineering, College of Life Sciences, Beijing Normal University, Beijing, 100875 China; 2 Key Laboratory of Animal Ecology and Conservation Biology, Institute of Zoology, Chinese Academy of Sciences, Beijing, China; 3 Department of Human Behavior, Ecology and Culture, Max Planck Institute for Evolutionary Anthropology, Leipzig, Germany; 4 Department of Natural Sciences, Faculty of Science and Engineering, Manchester Metropolitan University, Manchester, UK

**Keywords:** Sexual selection, Evolutionary theory

## Abstract

Existing sexual selection theory postulates that a sufficiently large variation in female fecundity or other direct benefits are fundamental for generating male mate choice. In this study, we suggest that, in addition to pre-pairing preferences, choosy males can also have different post-pairing behaviors, a factor which has been comparatively overlooked by previous studies. We found that both male preferences and female traits could evolve much more easily than previously expected when the choosy males that paired with unpreferred females would allocate more efforts to seeking additional post-pairing mating opportunities. Furthermore, a costly female trait could evolve when there was a trade-off between seeking additional mating and paternal care investment within social pair for choosy males. Finally, a costly male preference and a costly female trait might still evolve and reach a stable polymorphic state in the population, which might give rise to a high variability in male choice and female traits in nature. We suggest that male mate choice may be even more common than expected, which needs to be verified empirically.

## Abstract (web summary)

Lyu and colleagues use theoretical models to examine the relationship between post-pairing male behaviors and female trait evolution. They find that male mate choosiness can be surprisingly influential on the evolution of costly female traits, but also that costly male preference and costly female traits could evolve to a polymorphic equilibrium under certain conditions.

## Introduction

In sexually reproducing species, sex roles have traditionally been labeled as female choice and male competition^1,2^. Male mate choice first drew attention from research on species with reversed sex roles in courtship and mate choice^3^ whereby males evolve a strategy of providing the majority of parental care investment instead of females^4^. An increasing number of empirical studies have shown, however, that male mate choice also exists in species with traditional sex roles^5,6^. Theoretically, male preferences are selected against due to the high level of competition over attractive females^7–9^, with a sufficiently large variation in female fecundity and/or quality (i.e., female viability) necessary to overcome the competitive cost and generate male preferences with traditional sex roles^5–7,10^. The current evolutionary theory, therefore, suggests that the conditions required for the evolution of male mate choice are much more constrained than those for the evolution of female choice^8^.

Very few studies have examined the possibility of male preferences driving the evolution of costly female traits^11^. Additionally, it has been proposed that direct fitness benefits associated with female traits (e.g., high fecundity or quality) may be essential for the evolution of female traits in nature^7,12–16^. For example, males may show preferences for females with a large body size, which should be highly correlated with increased female fecundity^5,17^. Other empirical studies have suggested that signaling traits such as plumage coloration in birds^18,19^, coloration in fish^20^, or sexual swellings in primates^21^ that are related to male choice may directly indicate female quality. It is noteworthy that, in contrast to the ubiquitous female choice for male traits, there are very few empirical examples of male preferences for female traits, which we suggest may be related to the conventional mindset that a female trait can only evolve when it is correlated with fecundity or quality. In this study, we explore how the male preferences and female traits may evolve without such a limitation.

In previous sexual selection models, male mate choice has been widely assumed to take effect during the pre-pairing period, i.e., choosy males exhibit divergent mating tendencies for females with or without the preferred signaling traits^9^. In this case, although choosy males are more likely to mate with their preferred females, not everyone succeeds due to male−male competition^7^. Furthermore, the general assumption in those models has been that choosy males would show no difference in post-pairing behavior regardless of when they mate with preferred or unpreferred females. We suggest that this assumption is an unnecessarily restrictive condition applied to previous models. In many animals (e.g., in birds and mammals) the reproductive stage of the life cycle does not end at copulation as males still need to ensure their own sperm fertilizes the eggs and perhaps provide paternal care in order to acquire fitness^22^. However, males may be cuckolded during the post-pairing period, resulting in multiple paternity^23–25^. Thus, males may face a post-pairing trade-off between seeking additional mating and investment in breeding within social pair, including mate guarding^26^, providing incubation feeding^27^, vigilance^28^, and different forms of paternal care^22^. Some empirical studies have indicated that males within a population may vary extensively in additional mating efforts^29^, or even have the flexibility to take the initiative to vary their effort allocation between additional mating and reproduction within social pair^30^. This mechanism of post-pairing male choice-generating sexual selection for female traits has comparatively seldom been studied^31^ or considered by theoretical models that explore the evolution of male mate choice (but see refs. ^15,32^).

In this study, we develop population genetic models to demonstrate that male preferences and female mating signals could evolve and be much more easily maintained than previously expected if choosy males vary in both pre- and post-pairing behaviors. Specifically, we assume that when choosy males form pair bonds with the unpreferred females, the underlying instinctive preference would drive those males to allocate greater effort to seek additional mating post-pairing. We further show that a costly female trait could evolve from direct selection originating from a post-pairing trade-off between male strategies, and that a costly male preference can still evolve to a polymorphic equilibrium. Our models represent an important extension of existing theory, which highlights the possibility of underestimating the pervasiveness of male preferences and female traits in nature due to their detection being hindered by the polymorphism. Because only a portion of males would have preferences, detecting the preference would require a larger sample size and repeated measurements in e.g., binary choice experiments, which are commonly used to detect mating preference. Similarly, as only a portion of females would express the signal in the population, a relatively limited sample size or sampling bias along with measurement or treatment may significantly affect the empirical results (e.g.,^33^), although some types of signals (e.g., plumage or coloration) may be easy to detect regardless. Perhaps the biggest challenge would be when trying to detect both the signal and the preference while the nature of the signal is uncertain (e.g., some feature of a complex vocalization). Each signal would need to be repeatedly presented to multiple males.

## Results

### Trade-off between mate guarding and seeking additional mating

Unlike previous modeling studies on male mate choice (e.g., refs. ^7,9^), we include a post-pairing stage into our population genetic models that allows choosy males to have different strategies during this period. We assume that when a female chooses a male mate, reproduction occurs during which the male mate faces different post-pairing trade-offs. We first consider an intuitive trade-off between allocating effort to seeking additional mating and defending within-pair paternity (e.g., through mate guarding^24,34,35^ or territory defense^36^). In this model, males contribute nothing but gametes to the offspring (which can be interpreted as all males contributing equally in paternal care).

We assume two loci denoted P and T in our model. The first locus P determines the male preference, while T determines the expression of a female signaling trait. Specifically, we assume P_2_ males have a preference to mate with T_2_ females pre-pairing. However, it does not mean that all P_2_ males would mate with their preferred T_2_ females due to the competition with other males. And if T_1_ females choose to mate with P_2_ males, the preference for T_2_ females might drive those P_2_ males to reduce their investment in breeding within social pair (e.g., reducing their effort to mate guarding or paternal care as we explored in this study), which in turn enables them to have more effort to seeking additional matings.

We consider a pre-pairing life stage similar to previous models^7,9^. The life cycle starts by male courtship. We assume that males with the preference P_2_ allele are 1 + *a* times more likely to court preferred females (T_2_) than unpreferred (T_1_) females (at a ratio of (1 + *a*):1, respectively). All males compete for limited mating opportunities in the population depending on their courtship efforts (see Eqs. 4, 5 in “Methods”). For analytical tractability, we assume polygyny in our model to ensure all females having equal mating success, and the females choose their mate based on the male’s courtship effort. Empirical studies have indicated that polygynous species may also engage in EPCs, causing the males to face a severe risk of cuckoldry in nature (e.g.,^37,38^).

After mating, a post-pairing life stage occurs, during which both males and females may engage in EPCs. We assume the ratios of within-pair offspring produced by females of different genotypes are determined by their male mates’ mate guarding investment. Specifically, P_1_ males (i.e., without preferences) would allocate a fixed effort to mate guarding, by which their female mates would engage in EPCs also at a fixed probability (denoted by *α*). For those females that have EPCs, we further assume that they would produce a proportion *β* of extra-pair offspring. Therefore, the female mates of P_1_ males can produce extra-pair and within-pair offspring at expected proportions of *αβ* and 1 − *αβ*, respectively. For analytical simplicity, we use a parameter *θ* (instead of 1 − *αβ*) to represent the proportion of within-pair offspring that can be fertilized by P_1_ males, implying that their female mates would produce a proportion 1 − *θ* of extra-pair offspring in general. For P_2_ males, we assume they would have a variable post-pairing behavior that depends on the phenotypes of their social mates. When they mate with unpreferred T_1_ females, P_2_ males are prone to reduce their mate guarding effort to seek additional matings^15^, and as a result, can only fertilize a reduced proportion (*θ* − Δ*θ*) of within-pair offspring (see Eq. 6 in “Methods”). However, they would act in a similar way to P_1_ males when they mate with the preferred T_2_ females.

We also assume P_1_ males would allocate fixed effort to seek EPCs (denoted by *e*), so do P_2_ males when they mate with the preferred T_2_ females. For P_2_ males that mate with unpreferred T_1_ females, however, they can spend additional effort (e.g., longer time and/or more energy) to seek EPCs (denoted by *e* + Δ*e*), because of their reduced mate guarding investment. After within-pair and extra-pair copulations, females then begin to produce offspring. We delineate the full model construction processes and the detailed model analysis in the Methods.

**Fig. 1:**
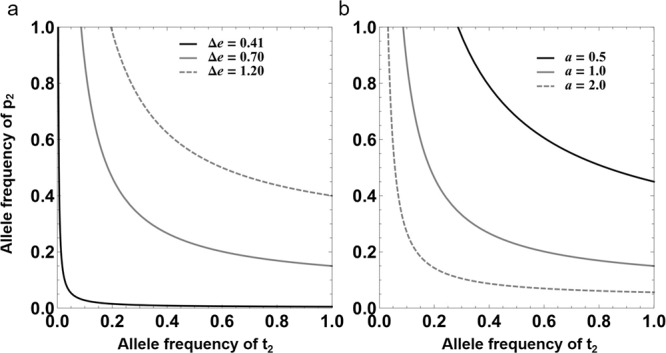
The stable internal equilibria of the two-locus models for the male preference P_2_ and female trait T_2_, allele frequencies when choosy males have a trade-off between mate guarding and seeking additional mating. **a** Effect of the relative increase in fitness gained from additional mating by P_2_ males ($\frac{\varDelta e}{e}$). The three lines in this graph represent different values of $\varDelta e$. **b** Effect of different strengths of male preference (*a*). The parameters behind those lines satisfy $\frac{\varDelta e}{e} > \frac{\varDelta \theta }{1-\theta }$, where $\frac{\varDelta e}{e}$ represents the relative increase in fitness gained from additional mating by P_2_ males when they mate with unpreferred T_1_ females, $\frac{\varDelta \theta }{1-\theta }$ represents the relative loss of paternity. For both panels, $e$ = 0.8, $\varDelta \theta =0.1$, and *θ* = 0.8. We set *a* = 1 in (**a**) and $\varDelta e=0.7$ in (**b**).

Contrary to previous modeling outcomes that consider only a pre-pairing life stage^7^, our model reveals a line of neutrally stable polymorphic equilibria (Fig. 1 and Supplementary Fig. 1) whereby male preference and the female trait could evolve and be maintained polymorphically when the following condition is met (detailed description of each symbol can be found in Table 1):

1 $$\frac{\varDelta e}{e} > \frac{\varDelta \theta }{1-\theta }$$

where $\frac{\varDelta e}{e}$ represents the relative increase in fitness gained from additional mating by P_2_ males when they mate with unpreferred T_1_ females, and$\frac{\varDelta \theta }{1-\theta }$ represents the relative loss of paternity. The equilibria of this model represents a balance between the competition for preferred T_2_ females, which selects against choosy P_2_ males^7^, and the selection for P_2_ males due to their potential for increased extra-pair paternity from additional mating to outperform within-pair paternity loss when they mate with unpreferred T_1_ females. Thus, an increased value of $\frac{\varDelta e}{e}$ generally has a positive effect on the equilibrium frequencies of both the P_2_ and T_2_ alleles (Fig. 1a).

**Table 1:** Detailed description of each symbol used in the models.

Symbols	Descriptions
*a*	Strength of male preference
*α*	The probability of engaging in EPCs for the female mates of P_1_ males
*β*	The proportion of extra-pair offspring produced by those females that have engaged EPCs
*θ*	Proportion of within-pair offspring fertilized by all P_1_ males, and P_2_ males when they mate with preferred T_2_ females, which is equal to 1 − *αβ*
Δ*θ*	Reduced proportion of within-pair offspring fertilized by P_2_ males when they mate with unpreferred T_1_ females
*e*	Effort allocated to seeking EPCs by all P_1_ males, and P_2_ males when they mate with the preferred T_2_ females
Δ*e*	Additional effort allocated to seeking EPCs by P_2_ males when they mate with unpreferred T_1_ females
*b*	Coefficient of the relative effect of male investment in reproduction within social pair, compared to female (i.e., 1)
*m*	Expected care effort of male parent
*δ*	Reduced proportion of care investment in breeding within social pair by P_2_ males when they mate with unpreferred T_1_ females
*s*_*f*_	Viability cost for T_2_ females that have the trait
*s*_*m*_	Viability cost for P_2_ males that have the preference

From condition (1), we can see that male preference (*a*) has no effect on determining the existence of stable polymorphic equilibria. However, a stronger male preference (*a*) can effectively hinder the evolution of male preference and female trait (i.e., resulting in lower equilibrium frequencies of P_2_ and T_2_, see Fig. 1b). Intuitively, when male preference (*a*) is stronger, the effect of direct negative selection on the male preference would be increased. Additionally, since choosy P_2_ males will be less likely to mate with unpreferred T_1_ females in this case (i.e., under a stronger male preference), it thus may lead to a much lower chance of benefitting from additional mating, further resulting in a reduced stable frequency of the male preference P_2_ allele (Fig. 1b).

Simulations indicated that initial frequencies of the P_2_ and T_2_ alleles determine the evolutionary outcome in this basic model (Supplementary Fig. 1). The near vertical evolutionary trajectories mean that very little evolution occurs in the female T_2_ trait allele because all females have the same mating success regardless of the locus T. The female trait evolves only because of the indirect selection from the genetic correlation between the female trait and the male preference^9^, as we find that a positive linkage disequilibrium between the loci P and T arises soon (Supplementary Fig. 2). Furthermore, we numerically confirmed that the linkage disequilibrium between the two loci at the line of polymorphic equilibria is always positive. The alleles P_2_ and T_2_ (and P_1_ and T_1_) are therefore associated along this line. Two mechanisms contribute to this association. First, P_2_ males show preferences to mate T_2_ females pre-pairing, resulting in nonrandom mating (i.e., it would be more likely to have P_2_ male-T_2_ female pairs than random mating). Second, when P_2_ males mate with unpreferred T_1_ females, they would reduce their guarding effort to look for extra-pair mating, resulting in a portion of offspring produced by the social pair (i.e., P_2_ male-T_1_ female) being replaced by extra-pair paternity (which could be with P_1_ males), while the P_2_ males get a chance to produce extra-pair offspring with T_2_ females through extra-pair mating. These two mechanisms thus enable P_2_ males to have a higher probability of producing offspring with T_2_ females than with T_1_ females, resulting in the positive linkage disequilibrium.

### Trade-off between paternal care investment and seeking additional mating

Here we also consider another possible post-pairing trade-off for males, which is between seeking additional mating and providing paternal care to ensure offspring survival and quality^22^. In this model, we assume males of all genotypes allocate the same effort to mate guarding, and thereby females will have the same probability to be involved in EPCs. For simplicity, we assume all females produce a proportion *θ* of within-pair offspring and a proportion 1 − *θ* of extra-pair offspring. For choosy P_2_ males, we assume they will reduce their care investment in reproduction within social pair by a proportion, *δ*, when they mate with unpreferred T_1_ females, which translates to a reduction in fecundity. As a trade-off, they can allocate more efforts (i.e., *e* + Δ*e*) to seeking additional mating (see “Methods”). In this case, the T_2_ female trait allele is more beneficial than the T_1_ allele due to the fecundity selection caused by male preference, i.e., T_1_ females (those without the trait) will suffer from a direct fitness loss when they mate with P_2_ males.

**Fig. 2:**
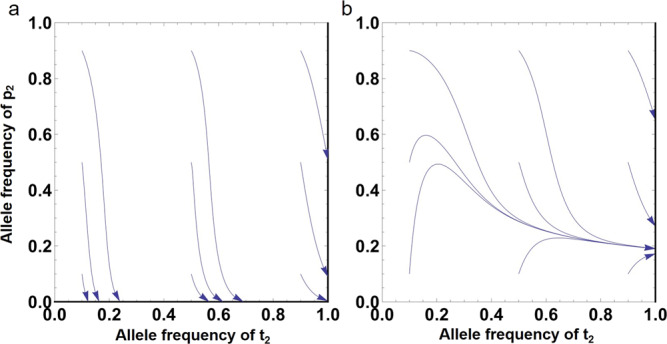
The stable equilibria for the male preference P_2_, and female trait T_2_ allele frequencies when choosy males have a trade-off between care investment and seeking additional mating. **a** When condition (2), i.e., $\frac{\varDelta e}{e} \, < \, \frac{b\delta \theta }{(1\,+\,b)(1\,-\,\theta )}$ is met, the model may evolve to an equilibrium point on the line of ${t}_{2}=1$ or on the line of ${p}_{2}=0$, depending on the initial frequencies. **b** When $\frac{\varDelta e}{e} > \frac{b\delta \theta }{(1+b)(1-\theta )}$ is met, it would always evolve to an equilibrium point on the line of *t*_2_ = 1. The arrowhead curves show the evolutionary trajectories under different initial states (*p*_2_ and *t*_2_ are set as 0.1, 0.5, and 0.9, respectively). We set $\varDelta e=0.1$ in (**a**) and $\varDelta e=0.8$ in (**b**). For all runs, the other parameters are: $e=0.8$, $a=1.5$, $\varDelta \theta =0.1$, $\delta =0.1$, $b=0.8$, and $\theta =0.8$. .

In this model, we find that the male preference locus always remains polymorphic when

2 $$\frac{\varDelta e}{e} > \frac{b\delta \theta }{(1+b)(1-\theta )}$$

is met. Moreover, the T_2_ female trait allele always becomes fixed within the population (Fig. 2b). Similar to the condition (1) in our first model, we can see that condition (2) also examines the fitness change in P_2_ males when paired with T_1_ females. The expression on the left-hand side represents the relative increase in extra-pair fitness due to increased effort. The expression on the right-hand side represents the relative loss in within-pair fitness due to reduced parental investment. When the condition is not met, both male preference and female trait can still evolve when the initial frequencies of the P_2_ and T_2_ alleles are relatively high (Fig. 2a). In both cases, a high frequency of preference is maintained only when both the preference and the trait alleles start at relatively high frequencies, with the preference locus evolving downward for the majority of starting conditions (Fig. 2). This is because under a higher frequency of the P_2_ allele, choosy P_2_ males not only face stronger competition pre-pairing, but also are more likely to gain a smaller amount of fitness from additional matings due to reduced paternal care investment by the social father, while their loss of within-pair fecundity remains constant. We also find that when the condition (2) is met, the frequency of the P_2_ allele would generally increase under a small initial value (Fig. 2b). In this case, relatively low competition for additional mating might enable choosy P_2_ males to gain enough fitness from EPCs to outcompete the selection on pre-pairing mate preference and the paternity loss of within-pair fecundity.

### Costly female trait can evolve in some conditions

Since expressing a signaling trait is costly to females^39^, we investigate whether such a costly female signal can also evolve through post-pairing behavior of choosy males. Specifically, we assume T_2_ females would suffer from a viability cost^7^, denoted by a coefficient *s*_*f*_. The life cycle starts with a viability selection on females (see “Methods”) and follows the same processes from the previous two models.

**Fig. 3:**
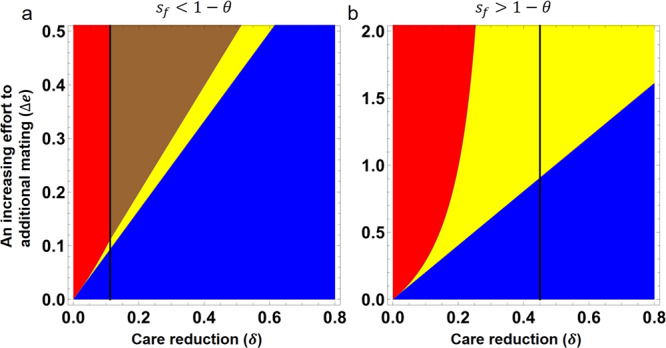
The conditions required for local stability of different cases of equilibrium when males with a preference trade-off between care investment and seeking additional mating, and the female trait is costly. The equilibria are given in the form of (*p*_2_, *t*_2_, D), where *p*_2_ and *t*_2_ represent the frequency of the allele P_2_ and S_2_ at each equilibrium, and D represents the corresponding linkage disequilibrium. Regions indicated in blue represent the conditions for local stability of (0, 0, 0). Equilibrium (1, 0, 0) is stable in the red color region. The region indicated in yellow represents the conditions required for local stability of an equilibrium point of ($\frac{\varDelta e(1\,+\,b)(1\,-\,\theta )\,-\,be\delta \theta }{\varDelta eb\delta }$, 0, 0). The brown region in the left panel (when ${s}_{f} < 1-\theta$ is satisfied) represents the conditions for a stable polymorphic equilibrium. The vertical black line shows the threshold value of *δ* when $\delta ={s}_{f}(1+1/b)$. In the region on the right side of this line, the model may also evolve to an equilibrium point on the line of ${t}_{2}=1$ depending on the initial frequencies (e.g., Supplementary Fig. 4a). Detailed conditions required for different equilibria stability can be found in the Supplementary Note 1. Numerical results of the internal equilibrium (allele frequencies and linkage disequilibrium between the two loci P and T) can be found in the Supplementary Fig. 5. We set ${s}_{f}=0.05$ and $\theta =0.7$ in (**a**), ${s}_{f}=0.2$ and $\theta =0.85$ in (**b**). The other parameters are: $b=0.8$, $e=0.8$.

Firstly, the female trait allele is always lost from the population when there is a trade-off between mate guarding and seeking additional matings for choosy males (Supplementary Fig. 3). Under the trade-off between care investment within social pair and seeking additional matings, however, both male preference and the costly female trait can still be maintained polymorphically under certain parameter values. Specifically, a relatively large reduction in care investment by P_2_ males that mate with T_1_ females (i.e., *δ*) and/or a low cost of female trait suffered by T_2_ females (i.e., *s*_*f*_) play fundamental roles in driving the evolution, i.e., requiring

3 $$\delta \, > \, {s}_{f}(1+1/b)$$

otherwise the female trait cannot evolve (Fig. 3; Supplementary Note 1). From the above condition, we can deduce that the female viability cost (i.e., *s*_*f*_) needs to be lower than $\frac{b\delta }{1\,+\,b}$, which represents the relative change of offspring produced in a single pair of T_1_ female and P_2_ male due to the reduction in paternal care. Under condition (3), there will always be a neutrally stable line of equilibria on *t*_2_ = 1 (see Supplementary Fig. 4a), enabling the evolution of both male preference and the female trait.

Furthermore, when the relative fitness increase of $\frac{\varDelta e}{e}$ is larger than $\frac{b\delta \theta }{(1\,+\,b)(1\,-\,{s}_{f}\,-\,\theta )}$ (and *s*_*f*_ < 1 − *θ* is met), the model may also reach a stable polymorphic equilibrium (the brown color region in Fig. 3a illustrates when the male preference and female trait are kept polymorphic in the population). In this instance, the evolutionary outcome is either an equilibrium point on the line of *t*_2_ = 1, or the polymorphic equilibrium point, depending on the initial frequencies (Supplementary Fig. 4a).

We find that the linkage disequilibrium between the P and T loci is always positive at the polymorphic equilibrium (Supplementary Fig. 5). It thus represents that the alleles P_2_ and T_2_ (and P_1_ and T_1_) are still associated under the trade-off between male paternal care and seeking additional mating. Note that in this model, P_2_ males would reduce their paternal care investment when they mate with unpreferred T_1_ females, resulting in a declined number of surviving offspring. Therefore, in combine with nonrandom mating pre-pairing, P_2_ males can also have a higher probability of producing offspring with T_2_ females, but a lower probability with T_1_ females than do P_1_ males, resulting in a positive linkage disequilibrium. In addition, numerical analysis indicates that the parameter space that allows the existence of the stable polymorphic equilibrium is larger when the strength of male preference (*a*) is higher (Supplementary Fig. 5).

#### Male mate choice with a viability cost can evolve

The costs of preferences have long been known to influence evolutionary outcomes in sexual selection models whereby some extra benefit from mate choice is required to enable the evolution of a costly female choice^40,41^. Here we also investigate whether male preference and female trait can still evolve when both are costly. For simplicity, we assume choosy P_2_ males also suffer from a viability cost^7^, denoted by a coefficient of *s*_*m*_ and that viability selection for the male preference happens before sexual selection (see “Methods”). The subsequent sexual selection and reproduction processes are the same as the previous basic models.

**Fig. 4:**
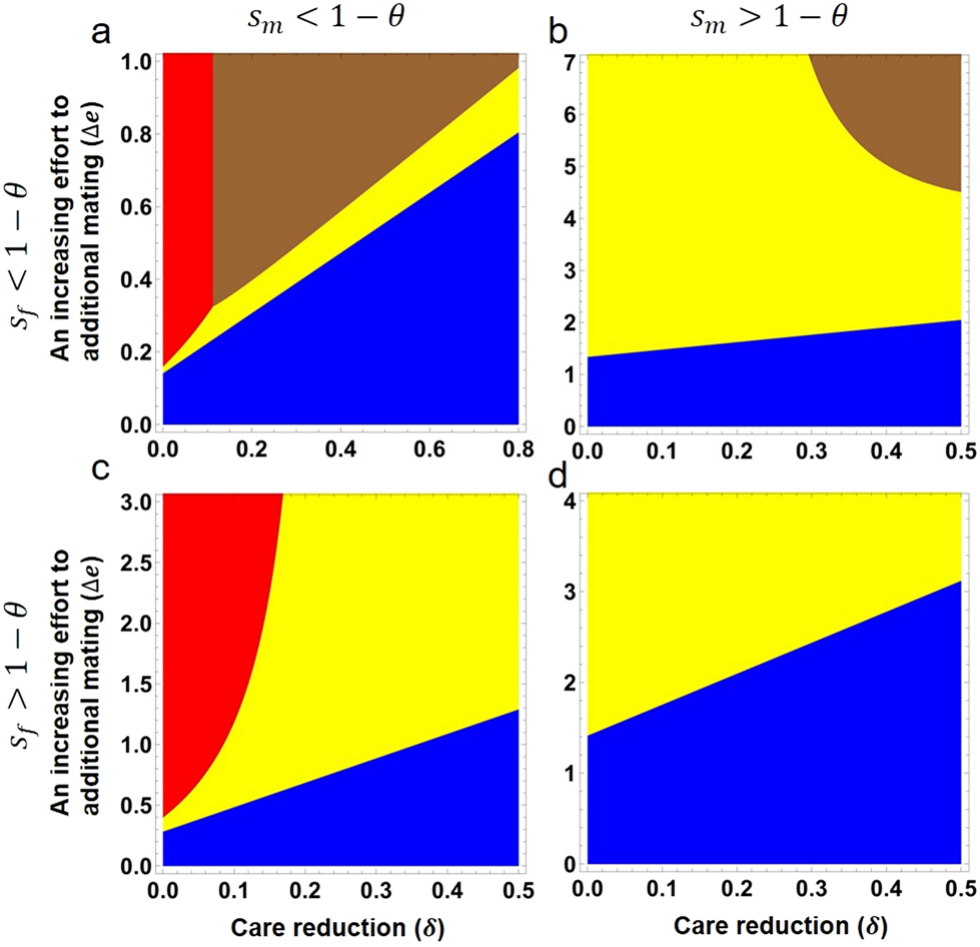
The conditions required for local stability of different cases of equilibrium when males with a preference trade-off between care investment and seeking of additional mating, and both male preference and female trait are costly. The color definitions for local stabilities are the same as Fig. 3. Regions indicated in blue, red and yellow represent the conditions for the local stability of equilibria (0, 0, 0), (1, 0, 0) and ($\frac{\varDelta e(1\,+\,b)(1\,-\,{s}_{m})(1\,-\,\theta )\,-\,e(b\delta \theta \,+\,{s}_{m}(1\,+\,b\,-\,b\delta \theta ))}{(\varDelta e(1\,-\,{s}_{m})\,-\,e{s}_{m})({s}_{m}(1\,+\,b(1\,-\,\delta ))\,+\,b\delta )}$, 0, 0), respectively. The stable polymorphic equilibrium exists in the brown color region in (**a**) and (**b**). Detailed conditions required for local stability of different equilibria can be found in Supplementary Note 2. Numerical results of the internal equilibrium (allele frequencies and linkage disequilibrium between the two loci P and T) can be found in the Supplementary Fig. 8. We set ${s}_{f}=0.05$, ${s}_{m}=0.05$ and $\theta =0.7$ in (**a**), ${s}_{f}=0.05$, ${s}_{m}=0.25$ and $\theta =0.8$ in (**b**), ${s}_{f}=0.17$, ${s}_{m}=0.05$ and $\theta =0.85$ in (**c**), and ${s}_{f}=0.15$, ${s}_{m}=0.15$ and $\theta =0.9$ in (**d**). The other parameters are: $b=0.8$, *e* = 0.8, $a=2$.

Intriguingly, we found that the model may still have a stable polymorphic equilibrium under the post-pairing trade-off between care investment within social pair and seeking additional mating (Fig. 4). Similar to the model with costly female traits described in the previous section, a relatively large reduction in care investment and a very low cost of the female trait are essential in generating a polymorphic equilibrium, i.e., requiring $\delta \, > \, {s}_{f}(1+1/b)$, and ${s}_{f} \, < \, 1-\theta$ (see Supplementary Note 2 for detailed conditions required for the stabilities of different equilibria). Furthermore, when the gain in fitness from additional mating is relatively large compared to the loss of within-pair paternity and viability costs, the polymorphic equilibrium would become the only stable point (illustrated by the brown color regions in Fig. 4a, b; and see Supplementary Note 2). When the cost of male preference (*s*_*m*_) is also limited, satisfying $(1-{s}_{f})(1-{s}_{m}) > \theta$, then polymorphic equilibrium can be achieved under a limited reduction in care (*δ*), and also under a small increase in effort toward additional mating (Δ*e*) (Fig. 4a, comparing to Supplementary Fig. 6), which should be biologically meaningful. Furthermore, either a higher proportion of paternity loss (i.e., under a smaller value of θ) or lower costs to male preference (*s*_*m*_) and/or female trait (*s*_*f*_) can effectively extend the parameter range that favors a stable internal equilibrium generally (Supplementary Fig. 7). As before, we find that the linkage disequilibrium between the P and T loci is still positive at the polymorphic equilibrium (Supplementary Fig. 8). Numerical analyses also indicate that a weak male preference (*a*) can have a dramatically positive effect on the equilibrium frequency of the allele T_2_ (Supplementary Fig. 8).

## Discussion

Following the inclusion of male post-pairing behavior into our sexual selection model, we show that male preferences and female traits can evolve even without direct benefits for female traits, such as the higher female fecundity and/or quality^11^. Furthermore, both costly male preference and costly female trait may evolve and remain polymorphic when choosy males face a trade-off between care investment within social pair and seeking additional mating^22^. These findings suggest that male mate choice might be more common than expected in nature.

Cuckoldry (or indeed the existence of extra-pair offspring) plays a fundamental role in driving the evolution of male mate choice in our model. EPCs have long been detected in different animal taxa and mating systems, occurring in approximately 90% of investigated avian species^42^, and several other animal taxa, such as mammals (e.g., refs. ^43–46^), including humans^47^. Therefore, considering post-pairing behavior in assessing the evolution of male mate choice should be relevant to a wide range of species. The commonly accepted principle of cuckoldry is that the social father loses some paternity but can also potentially gain some extra paternity from engaging in additional mating with females that are already paired^48^. Whether cuckoldry is advantageous or disadvantageous to males thus depends on net yields^15,32^.

In our models, the potential gain (and loss) are dependent on the frequencies of male preference and female trait alleles, which in turn leads to different evolutionary equilibria. On one hand, the total potential available fitness from EPCs in the population depends on the frequencies of the alleles P_2_ and T_1_, because choosy P_2_ males would either reduce mate guarding effort (resulting in an increased ratio of extra-pair offspring produced, i.e., from 1 − *θ* to $1-(\theta -\varDelta \theta )$ in the first trade-off) or paternal care (resulting in a smaller number of survival extra-pair offspring, i.e., from $(1+b)(1-\theta )$ to $[1+b(1-\delta )](1-\theta )$, see Eq. 8 in the second trade-off) when they mate with unpreferred T_1_ females. On the other hand, the extra-pair fitness gained by males of different genotypes is also frequency-dependent. For example, if the proportion of P_2_-T_1_ (male−female) mating types increases, the competition for additional mating would become more severe, because more males would allocate increased additional effort to seek EPCs.

In this study, we also show that a costly female trait can evolve when choosy males face a post-pairing tradeoff between care investment within social pair and seeking additional mating; in this case, T_1_ females, which do not have the trait, would suffer from a fitness loss when they choose P_2_ males. Thus there is the potential for the viability cost of the female trait to be offset by male choice. We suggest that even if the female trait is not correlated to direct fitness benefits, post-pairing male mate choice may drive the evolution of that trait. Furthermore, we note that relatively less costly traits (e.g., subtle characters) play essential roles in driving the evolution of female traits (see condition 3), which is consistent with several empirical studies on different animal taxa, in which female ornaments (or signals) have been reported to be relatively subtle^11^.

For example, during breeding seasons, female two-spotted gobies (*Gobiusculus flavescens*) have orange bellies^20^, and female spotted plateau lizards (*Sceloporus virgatus*) have orange throat patches^49^. Both traits have been empirically confirmed to be related to male choice. In contrast to the flashy colors commonly displayed in males of many other fish or lizard species, the female traits expressed by *G. flavescens* and *S. virgatus* are more subtle^11^. Given our model shows that female traits could be easy to evolve if it is not costly, a possible reason for these more subtle traits could be that they are costly. Given that females have to provide bulk of resources for developing offspring, a female may suffer more fecundity cost than a male when expressing the same trait^16^. Alternatively, female ornaments have also been explained as a genetically correlated by-product of sexual selection on males^39,50^. For instance, ornaments in female birds generally share the basic features of male ornaments in the same species, which may indicate a common genetic basis^51^. In this case, our modeling results highlight the possibility that males may easily evolve the preferences for these by-products, allowing females to overcome the costs for expressing the ornament traits instead of evolving towards sex-specific expression. This seems to suggest that the mating preferences of the two sexes may target the same ornament^52^, and that mutual mate choice may be present in sexually monomorphic species, which is not where researchers typically look for sexual selection. In reality, this pattern of subtle female ornamentation may have escaped our attention historically^31^, leading to a further underestimation of the pervasiveness of male preferences in driving the evolution of costly female traits.

The evolution of male preferences may be constrained by different costs^5^, such as the predation risk during mate searching and assessment, the inaccurate assessment of potential mates^10^, and a low probability of future matings^53^. In their sexual selection model, Servedio and Lande^7^ indicated that a viability cost to choosy males would accelerate the loss of the allele for male preference or prevent its spread when choosy males court more. Our results, however, reveal that both a costly male preference and costly female trait could evolve to a polymorphic equilibrium in the population (Supplementary Fig. 4b). We note that some empirical studies did find that both the abundance of female morphs and male preference varies among different populations (e.g.,^54^). This is consistent with our model prediction because different populations might evolve into various polymorphic equilibria. Further empirical investigation is required to verify how widespread this possibility may be.

It is noteworthy that our model hinges on the assumption that the pre-pairing male mate choice and the post-pairing guarding behavior or the paternal care investment are determined by the same locus. While it seems reasonable to us that when a male is paired with a unpreferred female, he would be more likely to seek out other mating opportunities. Several recent empirical studies on birds have found that supergenes can determine the reproductive morphs or behavioral phenotypes associated with mating, e.g., in ruff (*Philomachus pugnax*)^55,56^, and white-throated sparrow (*Zonotrichia albicollis*)^57,58^. We recommend further study focusing specifically on whether the pre-pairing choice and the post-pairing strategies are determined by the same locus, as well as theoretical studies on whether a linkage between the pre- and post-pairing behavior can evolve. Also worth noting is that we have conservatively assumed that females cannot receive benefits from EPCs in our models according to some recent studies (e.g.,^59,60^); however, if there are additional benefits for females^42^, it should further promote the evolution of male preferences and female traits in our model framework.

Our results extend the current understanding of the direction of sexual selection and suggests the possibility that male preferences and female traits are more widespread in nature, only awaiting to be tested empirically. It has been suggested that in many animals cuckoldry may be an important source of selection pressure on behaviors or morphologies in both sexes^15,32^, thus the mechanism proposed by our models may be relevant in many species. We suggest that understanding the evolutionary consequences of the effect of cuckoldry deserves more attention. Finally, we note that our models are framed focusing on male choice; the evolutionary dynamics of mutual mate choice considering both pre- and post-pairing behavior remains to be explored, as male and female mate choice may behave very differently in determining the evolutionary results^7^.

## Methods

### Basic models

We constructed a haploid two-locus population genetic model with non-overlapping generations to assess the evolution of male mate choice and a female signaling trait. One locus P determines a male preference, while the other, T determines a female trait. Each locus has two alleles, which results in four genotypes P_1_T_1_, P_1_T_2_, P_2_T_1_, and P_2_T_2_, with frequencies denoted by *x*_1_, *x*_2_, *x*_3_, and *x*_4_. Since P_2_ males would court their preferred females more vigorously, the proportion of effort spent in courtship by males of genotype *j* with females of genotype *i* can be derived as follows:

4 $${M}_{ij}=\frac{{x}_{i}{x}_{j}(1+da)}{{y}_{j}}$$

where *d* = 1if *i* is even (i.e., females that have the allele T_2_) and *j* is 3 or 4 (i.e., males that have the allele P_2_) and *d* = 0 otherwise, and$\,{\sum }_{ij}^{}{M}_{ij}=1$. Because males of different genotypes should only differ in their allocation of courtship effort, not the total amount of effort, for each male genotype *j* the proportion of effort spent on each female genotype should sum to the male genotype frequency, i.e., ${\sum }_{i}{M}_{ij}={x}_{j}$. We can then solve for the normalization term ${y}_{j}={\sum }_{i}{x}_{i}(1+da)$.

Subsequently, females choose their social mates among those courting males in proportion to the frequency and courtship effort of each male genotype in the population. As per previous models^7^, we assume that all females mate and have an equal mating rate. Then we have the proportion of each mating type as

5 $${F}_{ij}=\frac{{M}_{ij}}{{z}_{i}}.$$

where $${z}_{i}={\sum }_{j}{M}_{ij}/{x}_{i}$$.

### A post-pairing trade-off between mate guarding and seeking additional mating

During post-pairing stage, we firstly considered a trade-off between mate guarding (i.e., protecting within-pair paternity) and seeking additional mating for males. In this model, the proportions of within-pair offspring produced by females of genotype *i* that mate with males of genotype *j* (denoted by Θ_*ij*_) are determined by male guarding investment, thus

6 $${\varTheta }_{ij}=\theta -k\varDelta \theta,$$

where *k* = 1 if *j* = 3 or 4 and *i* is odd, otherwise *k* = 0 (see in the main text). Correspondingly, the efforts for seeking EPCs by males of genotype *j* that mate with females of genotype *i* are ${E}_{ij}=e+k\varDelta e$, where *k* =1 if *j* is 3 or 4 and *i* is odd, otherwise *k* = 0. We assume the fecundity benefits gained from EPCs by males of different genotypes are directly determined by their effort on seeking EPCs^15^. Thus, the proportions of extra-pair offspring sired by males of different genotypes in the population are

7 $${\rho }_{j}=\frac{{\sum }_{i}{F}_{ij}{E}_{ij}}{{\sum }_{ij}{F}_{ij}{E}_{ij}},$$

After combining the pre- and post-pairing life stages in our model, we obtain two 4 × 4 matrices representing the proportions of surviving within-pair (${O}_{ij}^{wp}$) and extra-pair offspring (${O}_{ij}^{ep}$) between each combination of parental genotypes, that is,

8a $${O}_{ij}^{wp}=\frac{{F}_{ij}{\varTheta }_{ij}{\phi }_{ij}}{{\sum }_{ij}{F}_{ij}{\phi }_{ij}},$$

8b $${O}_{ij}^{ep}=\frac{{\rho }_{j}{\sum }_{j}{F}_{ij}{\phi }_{ij}(1-{\varTheta }_{ij})}{{\sum }_{ij}{F}_{ij}{\phi }_{ij}}$$

where *ϕ*_*ij*_ represents the number of surviving offspring produced by a single pair in which the male has the *j* genotype and the female has the *i* genotype. Note that *ϕ*_*ij*_ is the same for all mated pairs in this model, and thus is set to a unit (i.e., 1). The proportion of total surviving offspring between each genotype is therefore ${O}_{ij}={O}_{ij}^{wp}+{O}_{ij}^{ep}$. Recombination and segregation follow mating (both within-pair and extra-pair) for two loci in haploids. Recombination rates are assumed for simplicity to be 0.5 between the two loci (i.e., free recombination). Recursion equations were used to compute the allele frequencies of *P*_2_ and *T*_2_, and the linkage disequilibria between the loci P and T in the next generation. Details of the recursion equations and numerical analyses of all model versions can be archived in *Mathematica* files on Zenodo (10.5281/zenodo.5717486).

### Another trade-off between paternal care and seeking additional mating

In our second post-pairing trade-off scenario for choosy males, the trade-off occurs between seeking additional mating and providing paternal care to the breeding within social pair. For analytical simplicity, we assume each female produces a proportion *θ* of within-pair offspring and a proportion 1 − *θ* of extra-pair offspring. During offspring production in this model, we assume the number of surviving offspring (*ϕ*_*ij*_) produced by females of genotype *i* with males of genotype *j* is directly determined by the investment from their social parents^61^: ${\phi }_{ij}=1+b{m}_{ij}$. In this equation, the first term (i.e., 1) represents the surviving offspring due to the care provided by the female parent, while the second term (i.e., *bm*_*ij*_) represents the surviving offspring due to the care provided by the male parent. We can treat *b* as a coefficient of the relative effect of male investment in reproduction within social pair, compared to female, and *m*_*ij*_ is the expected effort of male parent. Choosy P_2_ males will reduce their care investment when their social mates are unpreferred (i.e., T_1_ females), resulting in a lower level of paternal care efforts as ${m}_{ij}=1-\delta$, where *δ* represents the care investment reduction by P_2_ males in reproduction within social pair when they mate with unpreferred T_1_ females. In this case, T_1_ females that mate with P_2_ males would suffer from a fitness loss (i.e., their fitness is $1+b(1-\delta )$ instead of 1 + *b*) comparing to T_2_ females and other T_1_ females that mate with P_1_ males. The number of surviving offspring *ϕ*_*ij*_ of each mating type thus becomes:

9 $${\phi }_{ij}=1+b(1-k\delta )$$

where *k* = 1 if *j* = 3 or 4 and *i* is odd, otherwise *k* = 0.

Similarly to our first model, we assume P_2_ males that mate with T_1_ females can have additional effort (i.e., $e+\varDelta e$) to seek EPCs as a trade-off for the reduced paternal care investment. The other processes like courtship, pairing, recombination and segregation remain the same as our first model, i.e., calculating the proportions of courtship effort and mating types following the Eqs. 4, 5, respectively; calculating the proportions of extra-pair offspring sired by males of different genotypes using the Eq. 7; and finally calculating the offspring number using the Eq. 8. Note that we used the Eq. 9 as the surviving offspring number ${\phi }_{ij}$ in Eq. 8 in this model.

### Costly female trait

In the above models, the female trait is assumed to be costless^9^. For our next model, we would like to know whether male preferences and female traits can still evolve when such traits confer a cost to female viability^7^. Specifically, we assume that T_2_ females that have a trait would suffer from a viability cost (denoted by a coefficient of ${s}_{f} > 0$)^7^. Therefore, we have four updated genotype frequencies for females denoted as ${x}_{i}^{{\prime} }$ (*i* ranges from 1 to 4):

${x}_{i}^{\prime}=\frac{(1-{b}_{f}{s}_{f}){x}_{i}}{1-{b}_{f}{t}_{2}}$, where *b*_*f*_ = 1 when *i* is even, and *b*_*f*_ = 0 otherwise; *t*_2_ represents the allele frequency of the female trait. The viability selection happens before sexual selection and all other processes like sexual selection, recombination and segregation are the same as our previous basic models. The two trade-offs described in the above two subsections are both considered here.

### Costly male mate choice

In order to investigate the effect of a cost of male preference in our model, we assume that choosy P_2_ males would also suffer from a viability cost (denoted by a selection coefficient of ${s}_{m} > 0$) during their searching for or evaluating the mates^7^. Then, the frequency of each male genotype *j* before sexual selection would be

${x}_{j}^{\prime}=\frac{(1-{b}_{m}{s}_{m}){x}_{j}}{1-{b}_{m}{p}_{2}}$, where ${b}_{m}=1$ if $j=3$ or 4 (i.e., males have the P_2_ allele), otherwise ${b}_{m}=0$. The sexual selection, recombination, and segregation processes are the same as our basic models. As we have found that the male trade-off between mate guarding and seeking additional mating cannot give rise to the evolution of both male choice and costly female traits (see Supplementary Fig. 3), we only considered the trade-off between paternal care and seeking additional mating in this model.

### Reporting summary

Further information on research design is available in the Nature Research Reporting Summary linked to this article.

## Supplementary information

Supplementary information

Reporting Summary

## Data Availability

All material required to replicate this study is available in Zenodo, 10.5281/zenodo.5717486

## Code availability

The Mathematica code to replicate the analyses and figures has been deposited in Zenodo, 10.5281/zenodo.5717486.
