# Supplementary material for: The significant role of post-pairing male behavior on the evolution of male preferences and female traits
Source: Commun Biol. 2022 Jan 10;5:4. doi: 10.1038/s42003-021-02961-x (PMC8748628; doi:10.1038/s42003-021-02961-x)
Supplement: Supplementary file 1 — Supplementary information [file 42003_2021_2961_MOESM1_ESM.pdf]

Supporting Information for

The significant role of post-pairing male behaviour on the evolution of male preferences and female traits

Nan Lyu[1,2✉], D. Justin Yeh[3], Huw Lloyd[4], and Yue-Hua Sun[2]

[1]Ministry of Education Key Laboratory for Biodiversity and Ecological Engineering, College of Life Sciences, Beijing Normal University, Beijing 100875, China.

[2] Key Laboratory of Animal Ecology and Conservation Biology, Institute of Zoology, Chinese Academy of Sciences, Beijing, China.

[3] Department of Human Behavior, Ecology and Culture, Max Planck Institute for Evolutionary Anthropology, Leipzig, Germany.

[4] Department of Natural Sciences, Faculty of Science and Engineering, Manchester Metropolitan University, Manchester, United Kingdom.

✉email: nanlyu@bnu.edu.cn

ORCID ID for Nan Lyu: https://orcid.org/0000-0002-2544-6864

Supplementary Note 1. Equilibria under $s_f > 0$ and $s_m = 0$

Model outputs when males with a preference trade-off between care investment and seeking additional mating, and the female trait is costly (i.e., $s_f > 0$). The equilibria are given in the form of (p_2, t_2, D), where p_2 and t_2 represent the frequency of the P_2 and S_2 allele at each equilibrium, and D represents the corresponding linkage disequilibrium. The stabilities of different equilibria areas follow:

(1) (0, 0, 0) is locally stable if $\Delta e < \frac{eb\delta\theta}{(1+b)(1-\theta)}$;

(2) (1, 0, 0) is locally stable if $\Delta e > \frac{eb\delta\theta}{(1+b)(1-\theta)-b\delta}$, $\delta < (1-\theta)(1+1/b)$ and $\delta < s_f(1+1/b)$;

(3) The stability of (p_2^*, 0, 0) is complicated, where $p_2^* = \frac{\Delta e(1+b)(1-\theta)-be\delta\theta}{\Delta eb\delta}$. When the cost to the female trait is relatively limited, i.e., when $s_f < 1-\theta$ is satisfied, it is locally stable if

$\frac{eb\delta\theta}{(1+b)(1-\theta)} < \Delta e < \frac{eb\delta\theta}{(1+b)(1-\theta)-b\delta}$ and $\delta < s_f(1+1/b)$, or if $\frac{eb\delta\theta}{(1+b)(1-\theta)} < \Delta e < \frac{eb\delta\theta}{(1+b)(1-s_f-\theta)}$ and $\delta > s_f(1+1/b)$; when $s_f > 1-\theta$, it is locally stable if $\frac{eb\delta\theta}{(1+b)(1-\theta)} < \Delta e < \frac{eb\delta\theta}{(1+b)(1-\theta)-b\delta}$ and $\delta < (1-\theta)(1+1/b)$, or if $\Delta e > \frac{eb\delta\theta}{(1+b)(1-\theta)}$ and $\delta > (1-\theta)(1+1/b)$;

(4) Each point (p_2^*, 1, 0) on the line of $t_2 = 1$ with $p_2^* > \frac{(1+a)(1+b)s}{a(1+b)s+b\delta}$ is locally stable if $\delta > s_f(1+1/b)$;

(5) Numerical analyses show that an internal equilibrium can exist and be stable only if $\Delta e > \frac{eb\delta\theta}{(1+b)(1-s_f-\theta)}$ and $\delta > s_f(1+1/b)$ are satisfied.

Supplementary Note 2. Equilibria under $s_f > 0$ and $s_m > 0$

Model outputs when males with a preference trade-off between care investment and seeking additional mating, and both the male mate choice and female trait are costly (i.e., $s_f > 0$ and $s_m > 0$). We use the following functions for illustration:

$D_1 = \frac{(1+b)(1-s_m-\theta)}{b(1-s_m)}$, $D_2 = s_f(1+1/b)$ and $D_3 = \frac{(1+b)s_f s_m \theta}{b(1-s_m)(1-s_f-\theta)}$;

$E_1 = \frac{e(b\delta\theta+s_m(1+b-b\delta\theta))}{(1+b)(1-s_m)(1-\theta)}$, $E_2 = \frac{e(s_m(1+b-b\delta)+b\delta\theta)}{1-s_m+b(1-s_m(1-\delta)-\delta-\theta)-\theta}$ and $E_3 = \frac{b\delta e(b\delta\theta+s_m(1-s_f+s_f\theta+b(1-s_f+s_f\theta-\delta\theta)))}{(1+b)(b((1-s_m)\delta(1-s_f-\theta)-s_f s_m\theta)-s_f s_m\theta)}$.

The stabilities of different equilibria are as follow:

(a) When $s_f < 1-\theta$, $s_m < 1-\theta$ *and* $(1-s_f)(1-s_m) > \theta$ *(see Fig. 4a), we have*

$D_1 > D_2 > D_3 > 0$.

(0, 0, 0) is locally stable if $\Delta e < E_1$;

(1, 0, 0) is locally stable if $\Delta e > E_2$ and $\delta < D_2$;

The stability of (p_2^*, 0, 0) is complicated, where $p_2^* = \frac{\Delta e(1+b)(1-s_m)(1-\theta)-e(b\delta\theta+s_m(1+b-b\delta\theta))}{(\Delta e(1-s_m)-es_m)(s_m(1+b(1-\delta))+b\delta)}$.

It is locally stable if $E_1 < \Delta e < E_2$ and $\delta < D_2$, or if $E_1 < \Delta e < E_3$ and $\delta > D_2$.

Numerical analyses show that the internal equilibrium would always be the only stable equilibrium point if $\Delta e > E_3$, and $\delta > D_2$.

(*b*) *When* $s_f < 1 - \theta$, $s_m < 1 - \theta$ *and* $(1 - s_f)(1 - s_m) < \theta$ *(see Fig. S5), we have*

$D_3 > D_2 > D_1 > 0$.

(0, 0, 0) is locally stable if $\Delta e < E_1$;

(1, 0, 0) is locally stable if $\Delta e > E_2$ and $\delta < D_1$;

(p_2^*, 0, 0) is locally stable if $E_1 < \Delta e < E_2$ and $\delta < D_1$, or if $\Delta e > E_1$ and $D_1 < \delta < D_3$, or if $E_1 < \Delta e < E_3$ and $\delta > D_3$;

Numerical analyses show that the internal equilibrium would always be the only stable equilibrium point if $\Delta e > E_3$ and $\delta > D_3$.

(*c*) *When* $s_f < 1 - \theta$, $s_m > 1 - \theta$ *(see Fig. 4b), we have* $\mathrm{D}_3 > \mathrm{D}_2 > 0 > \mathrm{D}_1$.

(0, 0, 0) is locally stable if $\Delta e < E_1$;

(p_2^*, 0, 0) is locally stable if $\Delta e > E_1$ and $\delta < \mathrm{D}_3$, or if $E_1 < \Delta e < E_3$ and $\delta > \mathrm{D}_3$;

Numerical analyses show that the internal equilibrium would always be the only stable equilibrium point if $\Delta e > E_3$ and $\delta > \mathrm{D}_3$.

(*d*) *When* $s_f > 1 - \theta$, $s_m < 1 - \theta$ *(see Fig. 4c), we have* $\mathrm{D}_2 > \mathrm{D}_1 > 0 > \mathrm{D}_3$.

(0, 0, 0) is locally stable if $\Delta e < E_1$;

(1, 0, 0) is locally stable if $\Delta e > E_2$ and $\delta < \mathrm{D}_1$;

$(p_2^*, 0, 0)$ is locally stable if $E_1 < \Delta e < E_2$ and $\delta < \mathrm{D}_1$, or if $\Delta e > E_1$ and $\delta > \mathrm{D}_1$;

(e) When $s_f > 1 - \theta$, $s_m > 1 - \theta$ *(see Fig. 4d), we have* $\mathrm{D}_2 > 0 > \mathrm{D}_1 > \mathrm{D}_3$.

(0, 0, 0) is locally stable if $\Delta e < E_1$;

$(p_2^*, 0, 0)$ is locally stable if $\Delta e > E_1$.

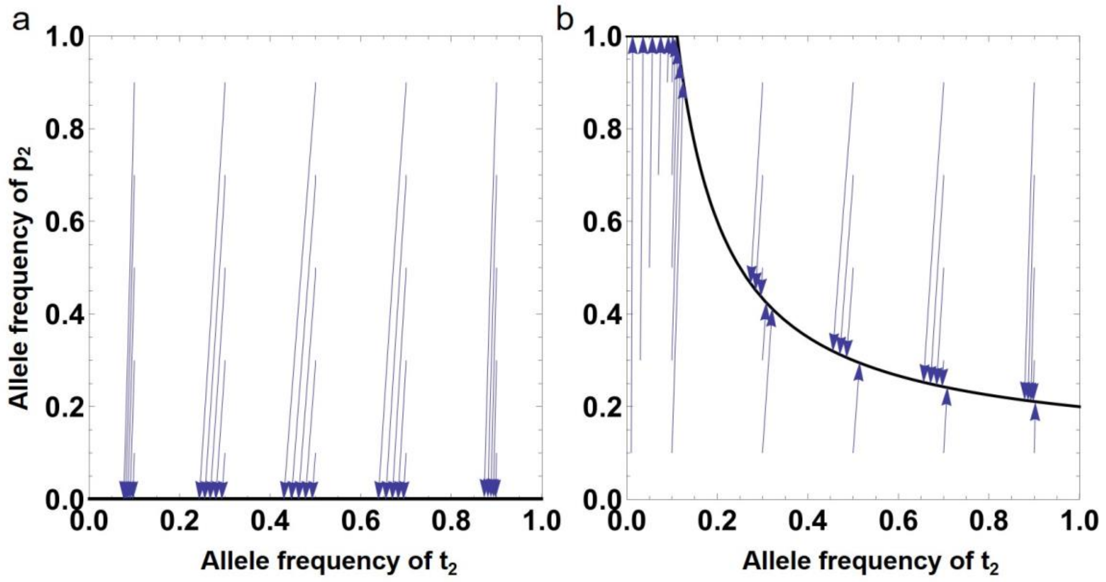

Supplementary Fig. 1 The stable equilibria for the male preference P_2 and female trait T_2, allele frequencies when males with a preference trade-off between mate guarding and seeking additional mating. (a) when the condition $\frac{\Delta e}{e} < \frac{\Delta \theta}{1-\theta}$ is met, male mate choice cannot evolve, (b) when $\frac{\Delta e}{e} > \frac{\Delta \theta}{1-\theta}$, the model may reach a polymorphic equilibrium point or an equilibrium point on the line of $p_2 = 1$, depending on the initial frequencies. The arrowhead curves show the evolutionary trajectories under different initial states with linkage equilibrium. We set $\Delta e = 0.2$ in (a) and $\Delta e = 0.8$ in (b). For all runs, the other parameters are: $e = 0.8$, $a = 1.0$, $\Delta\theta = 0.1$, $b = 0.8$, and $\theta = 0.8$.

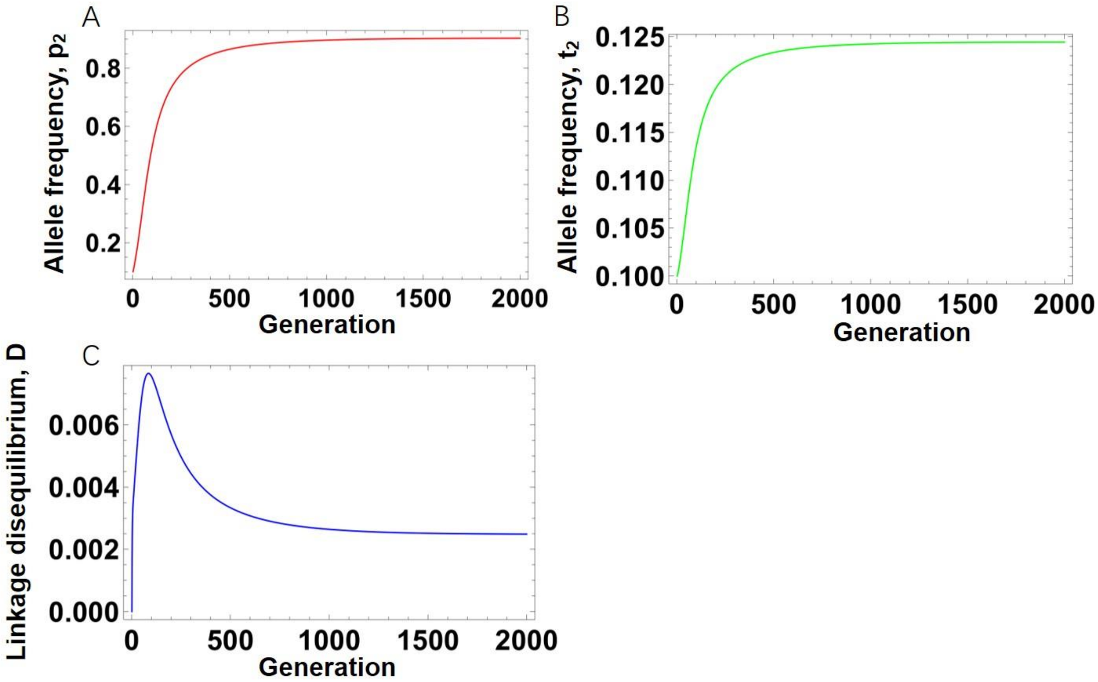

Supplementary Fig. 2 The example of dynamics of allele frequencies of p_2 (red curve) and t_2 (green curve) in (a) and (b) and linkage disequilibrium (D) of the two loci P and T (blue curve) in (c). The initial state is set as $p_2 = 0.1$ and $t_2 = 0.1$ with linkage equilibrium. The parameters are set as the same as in the Supplementary Fig. 1b.

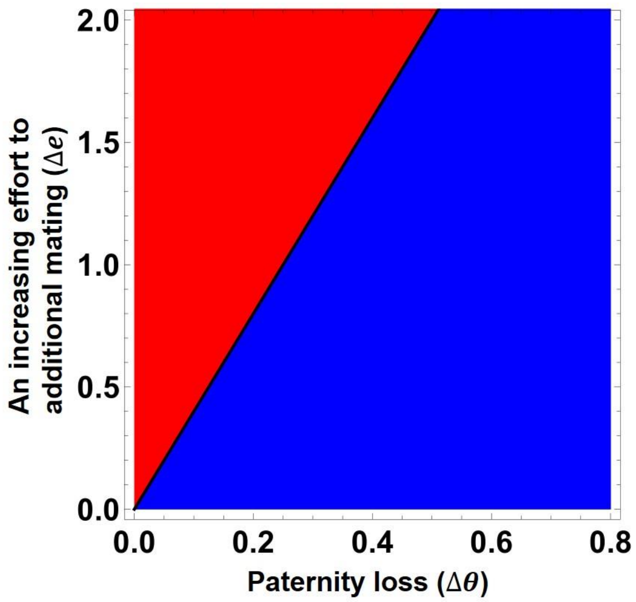

Supplementary Fig. 3 The conditions required for local stability of the two cases of equilibrium when males with a preference trade-off between mate guarding and seeking additional mating, and the female trait is costly. The equilibria are given in the form of (p_2, t_2, D), where p_2 and t_2 represent the frequency of the P_2 and S_2 alleles at each equilibrium, and D represents the corresponding linkage disequilibrium. Equilibrium (1,0,0) is stable in the red region, and equilibrium (0,0,0) is stable in the blue region. The oblique black line is $\Delta e = \frac{e\Delta\theta}{1-\theta}$. The parameters are set as: e = 0.8, $a = 1.5$, $b = 0.8$, and $\theta = 0.8$.

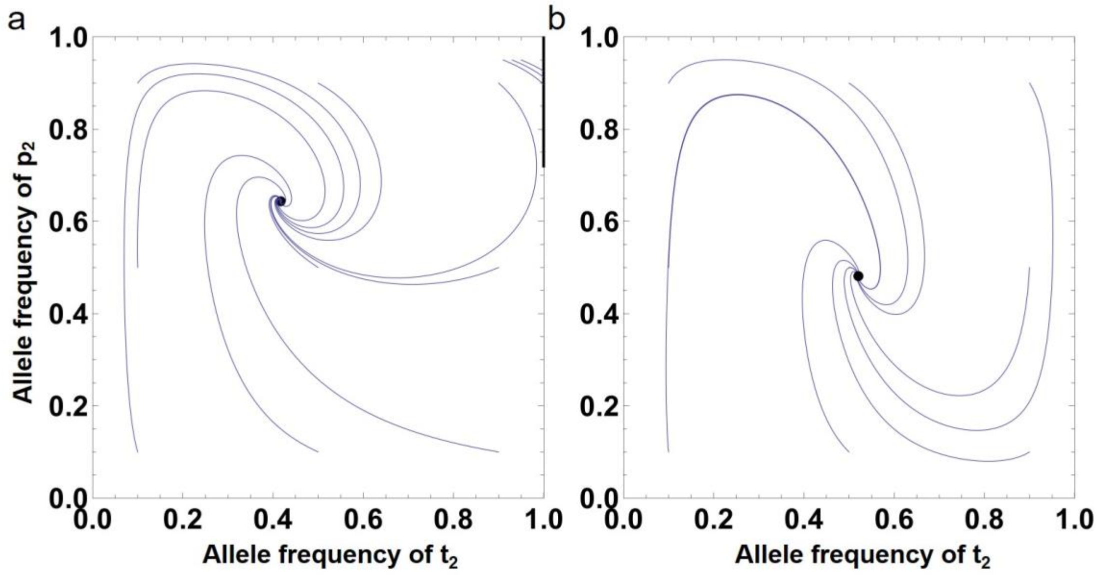

Supplementary Fig. 4 The evolutionary trajectory toward the stable internal equilibria when males with a preference trade-off between care investment and seeking additional mating. (a) When the female trait is costly, the model is bi-stable depending on the initial frequencies. (b) When both male mate choice and female trait are costly, it would always evolve to the polymorphic equilibrium. Parameters are set as: e = 0.8, $a = 1.0$, $\Delta e = 0.7$, $\delta = 0.2$, $s_f = 0.05$, $b = 0.8$, and $\theta = 0.7$ in (a). We set $s_m = 0.02$, $s_f = 0.02$ and $\delta = 0.12$ in (b).

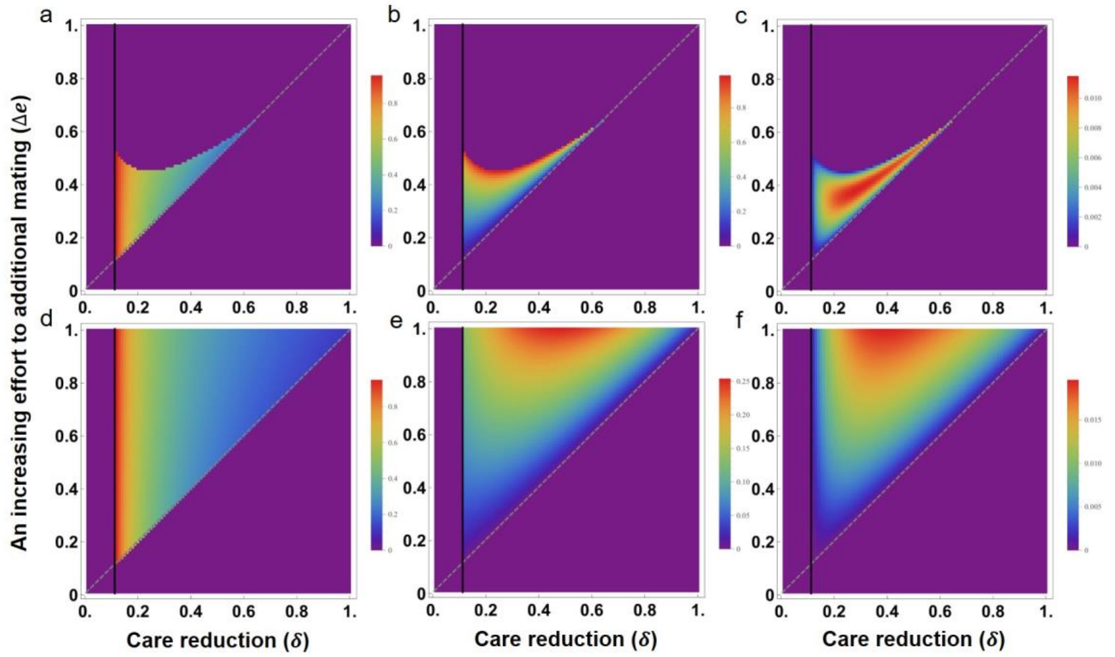

Supplementary Fig. 5 Numerical outputs of the stable internal equilibria for the male preference P_2 and female trait T_2, allele frequencies and linkage disequilibrium between the two loci P and T when males with a preference trade-off between care investment and seeking additional mating, and the female trait is costly. (a) and (d) represent the stable frequency of male preference, p_2, (b) and (e) represent the stable frequency of the female trait, t_2. (c) and (f) represent the linkage disequilibrium (D). The legends show the corresponding allele frequencies of different colors. We set $a = 0.5$ in (a), (b) and (c), $a = 2$ in (d), (e) and (f). The other parameters are set as: $e = 0.8$, $s_f = 0.05$, $b = 0.8$, and $\theta = 0.7$.

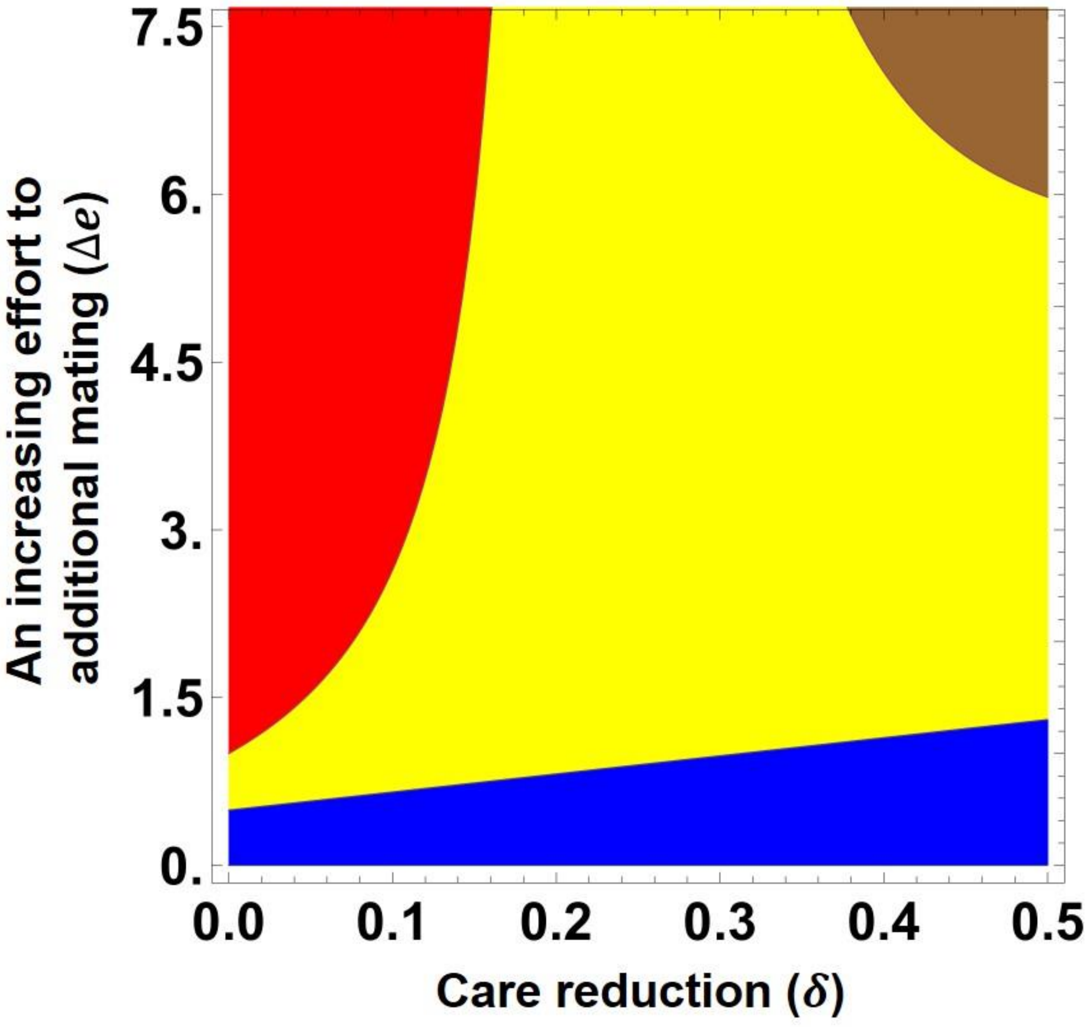

Supplementary Fig. 6 The conditions required for local stability of different cases of equilibrium when both male preference and female trait are costly when $s_f < 1 - \theta,\ s_m < 1 - \theta$ are satisfied, but $\theta > (1 - s_f)(1 - s_m)$. The color definitions for local stabilities are the same as in Figure 4. We set $s_f = 0.1,\ s_m = 0.1$ and $\theta = 0.82$. The other parameters are the same as in Figure 4a.

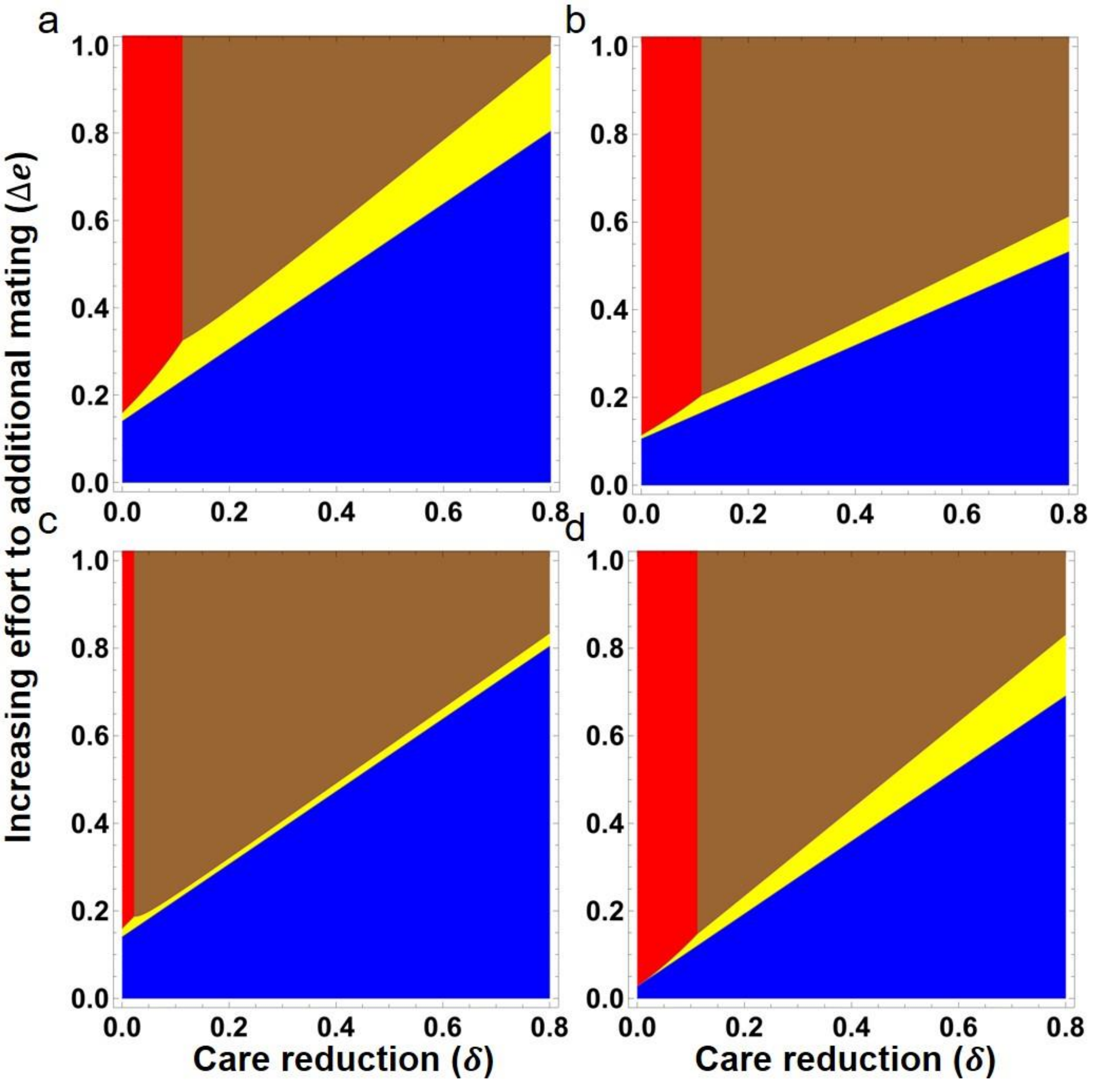

Supplementary Fig. 7 Either a higher proportion of paternity loss (i.e., under a smaller value of θ), or lower costs to male preference (s_m) and/or female trait (s_f) can effectively extend the parameter range that favours a stable internal equilibrium (i.e., brown region). (a) is the same graph as Figure 4a ($\theta = 0.7,\ s_f = 0.05,\ s_m = 0.05$). We set $\theta = 0.6$ in (b), $s_f = 0.01$ in (c), and $s_m = 0.01$ in (d). The other parameters are the same as we set in Figure 4a.

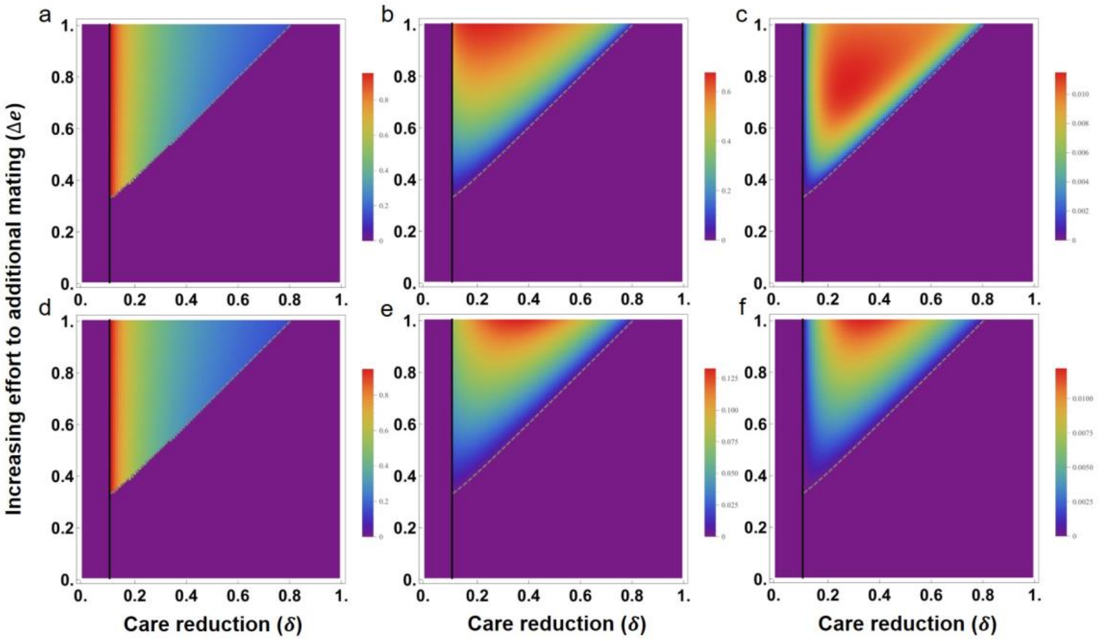

Supplementary Fig. 8 Numerical results of the stable internal equilibria for the male preference P_2 and female trait T_2, allele frequencies and linkage disequilibrium between the two loci P and T when males with a preference trade-off between care investment and seeking additional mating, and both male choice and female trait are costly. (a) and (d) represent the stable frequencies of male preference, p_2, (b) and (e) represent the stable frequencies of the female trait, t_2. (c) and (f) represent the linkage disequilibrium (D). The legends show the corresponding allele frequencies of different colors. We set $a = 0.5$ in (a), (b) and (c), and $a = 2$ in (d), (e) and (f). The other parameters are set as: $e = 0.8$, $s_f = 0.05$, $s_m = 0.05$, $b = 0.8$, and $\theta = 0.7$.
